# Prevalence, patterns, and predictors of patient-reported non-motor outcomes at 30 days after acute stroke: Prospective observational hospital cohort study

**DOI:** 10.1177/17474930231215660

**Published:** 2023-12-18

**Authors:** Hatice Ozkan, Gareth Ambler, Gargi Banerjee, Simone Browning, Alex P Leff, Nick S Ward, Robert J Simister, David J Werring

**Affiliations:** 1 UCL Queen Square Institute of Neurology, London, UK; 2 Hyper Acute Stroke Unit, National Hospital for Neurology and Neurosurgery, University College London Hospitals NHS Foundation Trust, London, UK; 3 Department of Statistical Science, University College London, London, UK; 4 MRC Prion Unit at UCL, Institute of Prion Diseases, London, UK

**Keywords:** Non-motor outcomes, patient-reported health outcomes, life after stroke

## Abstract

**Background:**

Adverse non-motor outcomes are common after acute stroke and likely to substantially affect quality of life, yet few studies have comprehensively assessed their prevalence, patterns, and predictors across multiple health domains.

**Aims:**

We aimed to identify the prevalence, patterns, and the factors associated with non-motor outcomes 30 days after stroke.

**Methods:**

This prospective observational hospital cohort study—Stroke Investigation in North and Central London (SIGNAL)—identified patients with acute ischemic stroke or intracerebral hemorrhage (ICH) admitted to the Hyperacute Stroke Unit (HASU) at University College Hospital (UCH), London, between August 1, 2018 and August 31, 2019. We assessed non-motor outcomes (anxiety, depression, fatigue, sleep, participation in social roles and activities, pain, bowel function, and bladder function) at 30-day follow-up using the Patient-Reported Outcome Measurement Information System-Version 29 (PROMIS-29) scale and Barthel Index scale.

**Results:**

We obtained follow-up data for 605/719 (84.1%) eligible patients (mean age 72.0 years; 48.3% female; 521 with ischemic stroke, 84 with ICH). Anxiety (57.0%), fatigue (52.7%), bladder dysfunction (50.2%), reduced social participation (49.2%), and pain (47.9%) were the commonest adverse non-motor outcomes. The rates of adverse non-motor outcomes in ⩾ 1, ⩾ 2 and ⩾ 3 domains were 89%, 66.3%, and 45.8%, respectively; in adjusted analyses, stroke due to ICH (compared to ischemic stroke) and admission stroke severity were the strongest and most consistent predictors. There were significant correlations between bowel dysfunction and bladder dysfunction (κ = 0.908); reduced social participation and bladder dysfunction (κ = 0.844); and anxiety and fatigue (κ = 0.613). We did not identify correlations for other pairs of non-motor domains.

**Conclusion:**

Adverse non-motor outcomes were very common at 30 days after stroke, affecting nearly 90% of evaluated patients in at least one health domain, about two-thirds in two or more domains, and almost 50% in three or more domains. Stroke due to ICH and admission stroke severity were the strongest and most consistent predictors. Adverse outcomes occurred in pairs of domains, such as with anxiety and fatigue. Our findings emphasize the importance of a multi-domain approach to effectively identify adverse non-motor outcomes after stroke to inform the development of more holistic patient care pathways after stroke.

## Introduction

Globally, stroke is the second most common cause of death and the third leading cause of disability-adjusted life years.^1^ Outcome measurement in stroke is dominated by motor impairments, communication, and mobility, often assessed using the modified Rankin scale (mRS), which does not fully capture the impact of key non-motor outcome domains on health-related quality of life after stroke.^2–7^ Furthermore, impairments not included in the mRS, such as post-stroke anxiety, depression, and fatigue, are associated with functional disability and premature death.^3,4^

Most previous studies investigated only individual non-motor domains, mainly in patients with ischemic stroke.^8–16^ We therefore aimed to (1) assess a full range of patient-reported non-motor outcome domains at 30 days after acute stroke; (2) identify the burden of adverse outcomes in multiple domains; (3) investigate correlations between non-motor outcome domains; and (4) determine baseline independent predictors (including ischemic stroke vs intracerebral hemorrhage, ICH) for each adverse non-motor outcome.

## Methods

### Study design and data source

The Stroke Investigation in North and Central London (SIGNAL) prospective hospital-based cohort study is based at the University College London Hospitals (UCLH) hyperacute stroke unit (HASU) which serves an ethnically diverse population of 1.6 million adults’ resident within five North Central London boroughs (Camden, Islington, Enfield, Harringay, and Barnet).

### Study population

We assessed all patients admitted consecutively with acute stroke between August 1, 2018 and August 31, 2019, for eligibility. Patients were included if they were aged ⩾ 18 years; resident in North Central London; had a clinical diagnosis of acute stroke (ischemic or hemorrhagic) validated by a consultant stroke physician and confirmed on brain imaging (computed tomography (CT), magnetic resonance imaging (MRI), or both) by a consultant neuroradiologist; and were able to provide complete data from two or more domains of the Patient-Reported Outcome Measurement Information System-Version 29 (PROMIS-29) patient-reported health outcome scale at 30-day follow-up (further details below). Sociodemographic and clinical characteristics including age, sex, ethnic origin, admission stroke severity (defined using the NIH Stroke Scale, NIHSS), previous medical history, medication history, discharge destination, and functional outcome at hospital discharge (mRS) were obtained from electronic health records.

We made every effort to include all eligible participants by providing additional supporting measures. We reduced patient burden for individuals who reported moderate to severe communication problems, cognitive impairment, or language difficulties by providing options to be given extra time, complete a postal questionnaire, or have the outcome measures translated or completed by a proxy responder. Proxy responders were eligible to assist individuals in completing the questionnaires if they were listed as a registered carer or next of kin (NOK) in the patients’ health records.

### Outcomes at 30-day follow-up

Patient-reported non-motor outcome scales were administered as part of routine care by clinically trained practitioners via telephone appointment. We used the PROMIS-29 v2.0 scale, which assesses seven health domains (physical function, anxiety, depression, fatigue, sleep disturbance, social participation, and pain) using four items per domain. Domains including anxiety, depression, fatigue, sleep disturbance, and pain capture individuals’ status in real time, asking about their experiences in the past 7 days. By contrast, the physical function and social participation domains capture status at the time of the initial stroke. Each PROMIS-29 domain score is standardized to the US general population on the T scale, with a mean of 50 and a standard deviation of 10; higher mean scores indicate worse outcomes. We considered an adverse non-motor domain to be present if the standardized domain score was ⩾ 55 (i.e. a score half a standard deviation worse than the general population).^11,15^

We measured bowel and bladder function using the Barthel Index. We considered individuals to have bowel or bladder dysfunction if they scored between 0 and 1, or if they had a urinary catheter at 30-day follow-up.

### Protocol approvals, registrations, and patient consent

The SIGNAL registry was approved by the UCL Hospitals NHS Foundation Trust Governance Review Board as a Service Evaluation (code: 5-201920-SE). Since the study data were collected as part of routine clinical care, the requirement for individual patient consent was waived.

### Statistical analysis

We summarized patient demographics and clinical characteristics with descriptive statistics. We compared continuous data using the Wilcoxon rank sum test, and categorical data with Pearson’s chi-squared tests. We recorded the clinical and sociodemographic characteristics of those patients who did not meet the inclusion criteria (Supplemental Material Table 1). We used histograms and q–q plots to understand the distribution of the data. We calculated the prevalence of adverse non-motor outcomes for each domain. We used the Pearson’s chi-squared test to compare the differences in prevalence of non-motor outcomes between ischemic stroke and ICH. We used the Benjamini–Hochberg false discovery rate procedure to guard against potential false-positive discoveries.^17^ We performed multivariable logistic regression analysis for individual non-motor outcome domains controlling for clinically relevant variables (age, sex, previous history of stroke) and characteristics significantly (p < 0.10) associated with each non-motor outcome in univariable analysis; The statistical tests were two-sided, and statistical significance was set at p < 0.05.

The characteristics of those reporting more than two or three adverse outcomes were compared to the total study sample (see Supplemental Material Table 3) using chi-squared test and t-test or the Kruskal–Wallis test as appropriate. We calculated Kappa statistics to quantify co-occurrence of non-motor outcome pairs. All statistical analysis were performed using Stata statistical software version 16.1.

## Results

### Patient characteristics

**Figure 1.**
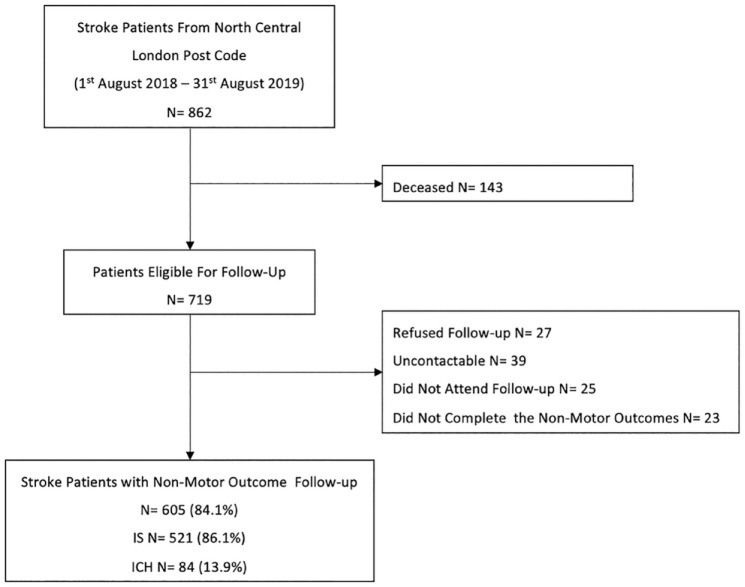
Patient selection flowchart. IS: ischemic stroke; ICH: intracerebral hemorrhage.

We included 605/719 (84%) of potentially eligible survivors (mean age 72.0 years; 48.3% female; 521 with ischemic stroke, 84 with ICH) (see Figure 1, and Table 1). The reasons for excluding 114 individuals were that: they declined follow-up; did not attend follow-up; were uncontactable; or were unable to provide the required non-motor data. Supplemental Material Table 3 provides detailed baseline characteristics of individuals excluded from our analysis.

**Table 1.** Demographic and clinical characteristic data for all patients and according to stroke type (ischemic stroke or ICH).

Characteristics	All stroke	IS	ICH	IS vs ICH p
	**605**	**521**	**84**	
Age range (IQR)	72.0 ± 14.9	72.2 ± 14.8	70.9 ± 15.8	0.4654
Female sex	292 (48.3%)	244 (46.8%)	48 (57.1%)	**0.103**
Ethnicity (n = 590), n (%)				
White	392 (66.4)	328 (63.0)	64 (76.1)	**0.057**
Asian	48 (8.1)	42 (8.1)	6 (7.1)	—
Black	28 (4.8)	26 (5.0)	2 (2.4)	—
Other	122 (20.7)	111 (21.9)	11 (13.25)	—
Medical history, n (%)				
Previous stroke/TIA	208 (34.4)	177 (33.9)	31 (36.9)	0.600
Hypertension	407 (67.9)	337 (65.4)	70 (83.3)	**0.001**
Congestive heart failure	27 (4.5)	22 (4.3)	5 (5.9)	0.489
Diabetes mellitus	166 (27.7)	143 (27.8)	23 (27.4)	0.942
AF	132 (21.8)	116 (22.3)	16 (19.1)	0.508
Dementia	9 (1.5)	6 (1.2)	3 (3.6)	0.617
Smoking history (n = 558)	208 (37.3)	180 (37.3)	28 (36.8)	0.933
Catheter	43 (7.1)	37 (5.8)	6 (6.7)	0.539
Medication history, n (%)				
Thrombectomy	32 (5.3)	32 (5.3)	0	—
Thrombolysis	124 (20.5)	124 (20.5)	0	—
Antiplatelet	340 (56.2)	327 (62.8)	13 (15.5)	**<0.001**
Anticoagulant	142 (23.5)	133 (25.5)	9 (10.71)	**0.003**
Antihypertensive	468 (77.7)	393 (75.9)	75 (80.3)	**0.006**
Statin	260 (42.9)	221 (42.4)	39 (46.4)	0.491
Antidepressants	23 (3.8)	19 (3.6)	4 (4.8)	0.739
Clinical outcomes median (range)				
Pre-morbid mRS	0 (0–1)	1 (0–1)	1 (0–2)	**0.0225**
Admission NIHSS	4 (2–8)	5.8 (2–9)	6.3 (4.5–12.5)	**0.0374**
Discharge mRS	3 (1–4)	1 (1–4)	3 (1–5)	**0.0426**
30-day mRS	2 (1–3)	1 (1–3)	2 (1–4)	**0.0235**
Time to follow-up	32.4 (28–36)	31.8 (26–34)	32.1 (29–36.3)	0.5837
Discharge location (n = 579), n (%)				
Home with ESD	141 (24.4)	127 (25.6)	14 (16.9)	**<0.001**
ASU	277 (47.8)	229 (46.2)	48 (57.8)	—
Care home	5 (0.9)	1 (0.2)	4 (4.8)	—
Home no ESD	156 (26.9)	139 (28.0)	(20.5)	—
Proxy response (N, %)	31 (5.1)	22 (4.2)	9 (10.7)	0.048

Significant differences in results are highlighted in bold.

IS: ischemic stroke; ICH: intracerebral hemorrhagic stroke; IQR: interquartile range; TIA: transient ischemic attack; AF: arterial fibrillation; NIHSS: NIH stroke scale score; mRS: modified Rankin Scale; ESD: early supported discharge; ASU: acute stroke unit.

We provided additional supporting measures to facilitate data completion in 51/605 (8.4%). Of these 51 patients who needed additional support, 9 had a recorded dementia diagnosis, 17 had moderate–severe memory problems, and 13 had severe communication problems, while 12 were non-English speakers. Regarding the additional support provided, 27/51 (52.9%) needed direct proxy assistance (from a registered carer or next of kin) to complete outcomes, 4 (7.8%) required proxy assistance to translate, and 20 (39.2%) required extra time either during the assessment or by being sent a postal pack.

### Prevalence of adverse non-motor outcomes

**Figure 2.**
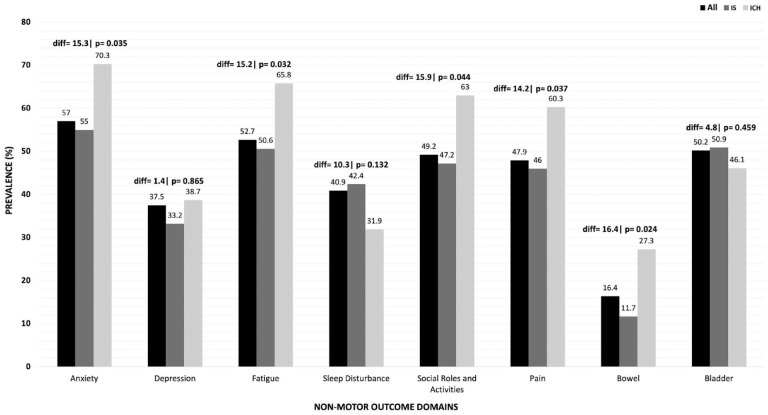
Prevalence of adverse non-motor outcomes at 30-day follow-up for all patients and according to stroke type (ischemic stroke or ICH). Adverse non-motor outcome prevalence at 30-day follow-up after acute stroke measured by six domains of the PROMIS-29 and the BI subscales for bowel and bladder function. diff = absolute percentage difference in adverse non-motor outcome domain between IS and ICH (two independent sample *t*-test). IS: ischemic stroke; ICH: intracerebral hemorrhage.

The most common adverse non-motor outcomes were anxiety (57.0%), fatigue (52.7%), impaired bladder function (50.2%), reduced participation in social roles and activities (49.3%), and pain (47.9%) (Figure 2). Compared to patients with ischemic stroke, the following adverse non-motor outcomes were significantly more common in patients with ICH: anxiety (difference 15.3%, 95% CI 0.03–27.4%); fatigue (difference 15.2%, 95% CI 0.02–19.3%); reduced participation in social roles and activities (difference 15.9, 95% CI 0.04–28.1%); pain (difference 14.2%, 95% CI 0.02–26.5%); and impaired bowel function (difference 16.4%, 95% CI 0.04–21.1%) (Figure 2; Supplemental Material Table 2).

Data from the multivariable analysis are shown in Table 2. Compared with patients with ischemic stroke, patients with ICH had significantly higher adjusted prevalence ratios for anxiety (adjusted odds ratio (OR) 1.83; 95% CI 1.01–3.12), fatigue (OR 1.80; 95% CI 1.00–3.24), reduced social participation (OR 1.72; 95% CI 1.02–3.19), pain (OR 1.93; 95% CI 1.14–3.41) and bowel dysfunction (OR 3.72; 95% CI 1.91–7.2). Other characteristics associated with adverse non-motor outcomes in multiple domains were age > 60 years; female sex; previous history of stroke or TIA; stroke severity on admission; ethnic origin; and discharge mRS score 4–5.

**Table 2.** Multivariable analysis of baseline factors associated with adverse non-motor outcomes.

		Adjusted OR [95% CI] p-value, IS vs ICH							
		Non-motor outcomes							
		Anxiety	Depression	Fatigue	Sleep disturbance	Social roles and activities	Pain	Bowel	Bladder
Stroke type (ICH vs IS)		**1.8 [1.0–3.1]** 0.035	0.9 [0.5–1.7] 0.839	**1.8 [1.0–3.2]** 0.042	0.9 [0.5–1.6] 0.662	**1.7 [1.0–3.2]** 0.046	**1.9 [1.1–3.4]** 0.030	**3.7 [1.9–7.2]** <0.001	0.8 [0.5–1.3] 0.345
Age		**0.5 [0.3–0.8]** 0.006	1.0 [0.6–1.6] 0.991	1.1 [0.7–1.8] 0.596	1.1 [0.7–1.8] 0.701	**1.7 [1.0–2.8]** 0.039	**2.3 [1.4–2.0]** 0.001	1.5 [0.7–3.0] 0.255	**1.5 [1.0–2.4]** 0.049
Female sex		0.9 [0.6–1.3] 0.461	1.0 [0.7–1.4] 0.951	**1.4 [1.0–1.9]** 0.054	1.3 [0.9–1.9] 0.168	1.1 [0.7–1.6] 0.678	**1.7 [1.2–2.5]** 0.004	1.3 [0.8–2.2] 0.296	**1.9 [1.3–2.7]** 0.001
Non-White ethnicity		0.9 [0.6–1.3] 0.431	**1.7 [1.1–2.4]** 0.011	1.1 [0.7–1.5] 0.773	**1.7 [1.2–2.5]** 0.006	1.0 [0.7–1.5] 0.927	0.8 [0.5–1.1] 0.164	0.9 [0.5–1.6] 0.853	**0.3 [0.2–0.4]** <0.001
Previous stroke/TIA		0.8 [0.5–1.1] 0.202	1.3 [0.9–1.9] 0.222	**1.5 [1.0–2.1]** 0.039	**1.6 [1.1–2.3]** 0.021	**4.2 [2.8–6.4]** <0.001	1.2 [0.8–1.7] 0.475	**3.0 [1.8–5.0]** <0.001	0.9 [0.6–1.4] 0.706
Hypertension		0.8 [0.6–1.2] 0.376	1.0 [0.6–1.4] 0.726	0.8 [0.5–1.1] 0.151	1.1 [0.7–1.6] 0.622	1.2 [0.8–1.8] 0.471	1.1 [0.8–1.7] 0.524	1.4 [0.8–2.6] 0.243	0.8 [0.5–1.2] 0.307
Admission NIHSS		**1.4 [1.0–2.1]** 0.031	**1.5 [1.0–2.3]** 0.018	0.9 [0.6–1.4] 0.715	1.2 [0.8–1.8] 0.474	**1.7 [1.1–2.7]** 0.016	1.2 [0.8–1.9] 0.398	**3.3 [1.9–6.1]** <0.001	**2.0 [1.3–3.0]** 0.003
Antiplatelet		0.9 [0.6–1.4] 0.741	0.8 [0.5–1.1] 0.200	0.8 [0.6–1.2] 0.253	1.4 [0.9–2.0] 0.177	0.9 [0.6–1.3] 0.429	1.2 [0.8–1.7] 0.418	0.6 [0.4–1.1] 0.101	1.3 [0.9–2.0] 0.140
Discharge mRS (0–1 reference)	mRS 2–3	0.8 [0.5–1.3] 0.359	**0.3 [0.2–0.8]** 0.013	1.1 [0.7–1.8] 0.667	1.1 [0.7–1.9] 0.138	**2.5 [1.2–5.7]** 0.018	1.0 [0.6–1.7] 0.920	1.6 [0.8–1.9] 0.751	1.0 [0.7–2.1] 0.516
mRS 4–5		1.1 [0.6–2.0] 0.715	**0.6 [0.4–0.9]** 0.010	1.0 [0.5–1.7] 0.897	0.8 [0.5–1.4] 0.472	**4.4 [1.4–6.3]** 0.009	1.2 [0.4–2.1] 0.528	**1.3 [1.1–3.0]** 0.016	**1.5 [1.0–2.4]** 0.048
Discharge location ASU		0.8 [0.4–1.3] 0.325	1.01 [0.6–1.8] 0.982	1.1 [0.6–1.6] 0.842	1.1 [0.7–1.9] 0.638	1.1 [0.6–2.1] 0.648	**1.4 [1.1–1.8]** 0.040	**1.9 [1.4–3.0]** 0.051	**1.7 [1.0–3.1]** 0.015
Care home		0.9 [0.5–1.5] 0.633	0.7 [0.4–1.3] 0.293	0.9 [0.6–1.6] 0.833	1.2 [0.7–2.2] 0.495	**2.3 [1.2–4.4]** 0.027	0.9 [0.7–1.9] 0.685	1.0 [0.5–2.2] 0.900	1.5 [0.6–2.5] 0.516

Reference groups: age < 60 years; male sex; white ethnic origin; discharge mRS 0–1; discharge destination home.

IS: ischemic stroke; ICH: intracerebral hemorrhagic stroke; NIHSS: NIH stroke scale score; mRS: modified Rankin Scale; ASU: acute stroke unit. Odds ratios for significant (P value <0.05) results are highlighted in bold.

### Prevalence of adverse non-motor outcomes in multiple domains

**Figure 3.**
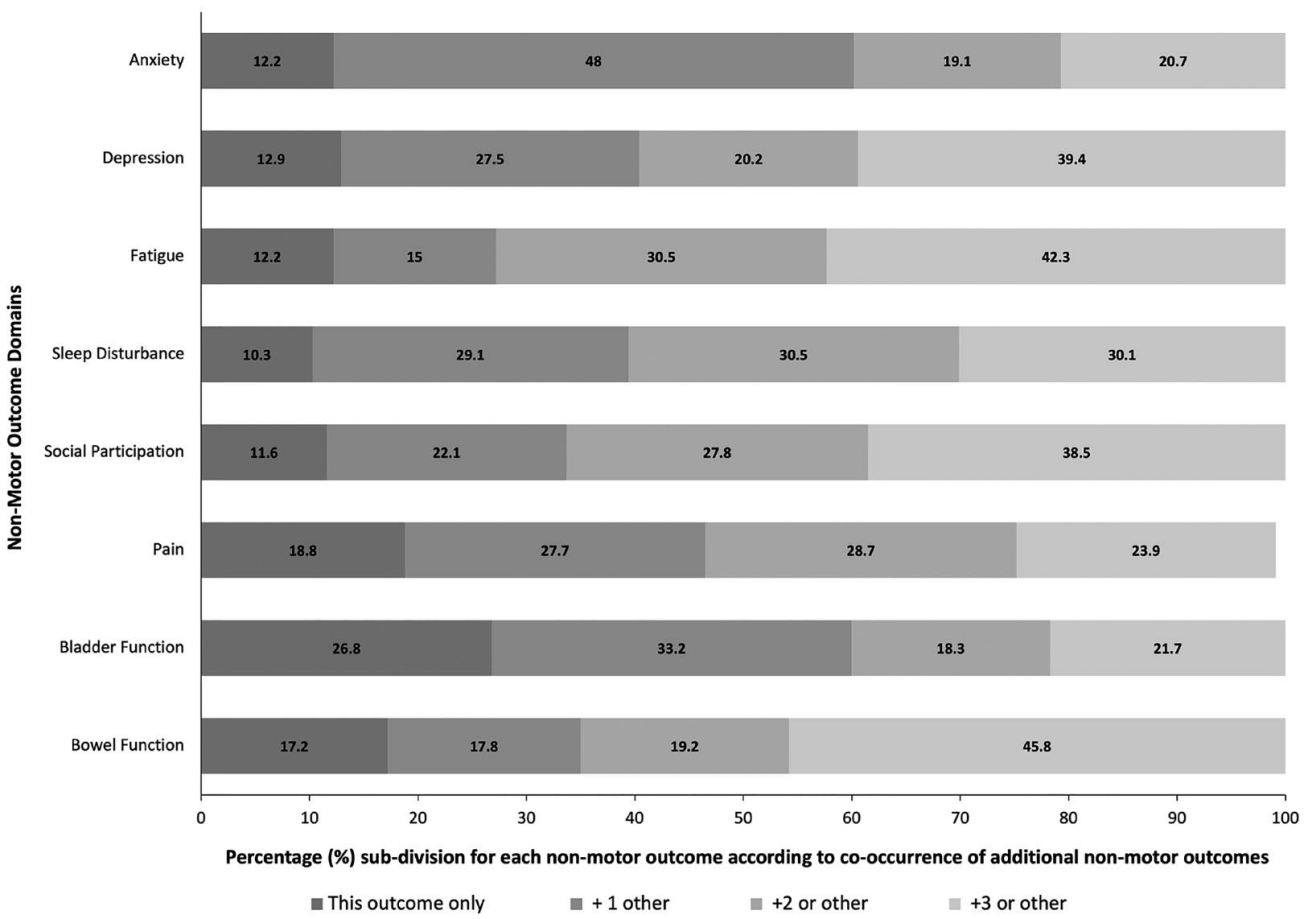
Co-occurrence of non-motor outcome domains.

The prevalence of ⩾1, ⩾2, and ⩾3 adverse non-motor outcomes were 88.4%, 66.3%, and 45.8%, respectively. Figure 3 shows how frequently each non-motor health domain outcome was associated with one, two, or three co-occurring outcomes. All non-motor domains were frequently associated with other co-occurring adverse outcomes. Compared to the total study sample, those reporting multiple (> 2) adverse non-motor outcomes had a higher stroke admission NIHSS (median = 7 vs 4, p = 0.0450) and hospital discharge mRS score (median 2 vs 1, p = 0.0230) (Supplemental Material Table 4).

**Figure 4.**
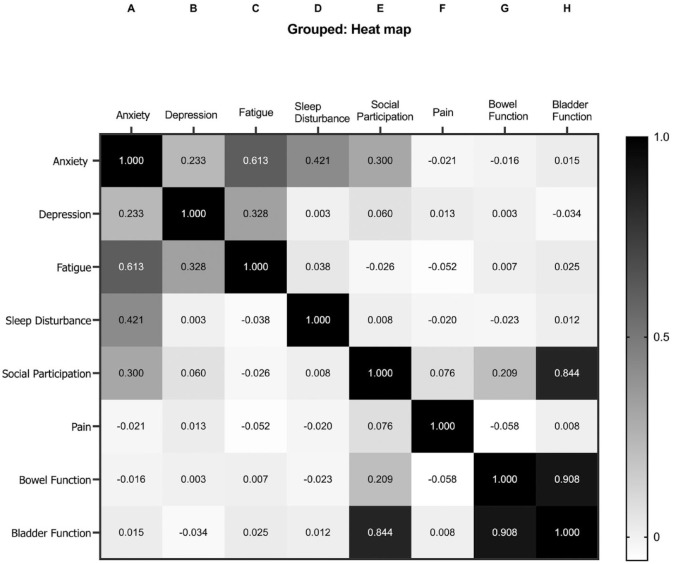
Correlations between pairs of non-motor outcome domains. *Kappa analysis to show proportion of agreement on outcome overlap beyond the observed prevalence identified by chance.

Correlations between each non-motor domain on PROMIS-29 and Barthel Index are summarized in Figure 4. There was substantial correlation for anxiety with fatigue (κ = 0.613); reduced social participation with bladder dysfunction (κ = 0.844); and bowel dysfunction with bladder dysfunction (κ = 0.908).

## Discussion

We found that adverse patient-reported non-motor outcomes were extremely common in people following acute stroke at 30 days: 88.4% of the evaluated stroke survivors reported at least one adverse non-motor outcome, 66.3% two or more, and 45.8% three or more. The health domains most affected were anxiety (57.0%), fatigue (52.7%), impaired bladder function (50.2%), and reduced participation in social roles and activities (49.3%). Most health domains were more likely to be affected after ICH compared to ischemic stroke, even after adjusting for confounding factors including stroke severity. We identified pairs of co-occurring domains, such as bowel dysfunction and bladder dysfunction, and reduced social participation and bladder dysfunction. Our findings have potential clinical implications: first, they show high burden of patient-reported non-motor outcomes; second, they underline a substantial unmet patient-reported healthcare need requiring the development of appropriate multidisciplinary specialist comprehensive multi-domain screening and care delivery pathways; third, they highlight those at highest risk, for example, people with stroke due to ICH; fourth, the design of future care pathways to address adverse patient-reported non-motor outcomes may be informed by our findings of associations between these outcomes (e.g. anxiety and fatigue) .

Our findings are in line with other, predominantly single-domain studies,^5–9,12,15,17–25^ including some which suggest that even after complete motor recovery, deficits in non-motor domains, such as anxiety, depression, fatigue, pain, bowel, and bladder dysfunction, may prevent a return to independent living.^12,13,15^ Our analysis extends these findings by providing new data for all key domains, and predictive factors, in the same cohort; few previous studies investigated all these domains in the same population.^14,15,17,25^

We found that anxiety affected 57% of patients and was associated with ICH (compared to ischemic stroke), age < 50 years and worse admission stroke severity. A previous meta-analysis found persistent anxiety in 38–76% of patients but did not investigate predictive factors.^3,12^ Small cohort studies suggest that ICH volume, lobar location, and MRI findings of cerebral amyloid angiopathy (CAA) predict anxiety after stroke,^26^ but further larger studies are needed in ICH subgroups.

Our findings that fatigue and depression are common (affecting 52.7% and 36.5% of patients, respectively) are consistent with previous reports describing fatigue in 42–53% (between 1 and 6 months after stroke) and depression in 24–39% (between 3 months and 5 years).^3,12,21,26^ We found that fatigue is more prevalent after ICH than ischemic stroke (65.8% vs 50% of patients, respectively), in line with a meta-analysis of small studies that found that ICH stroke survivors had nearly double the prevalence of fatigue compared to ischemic stroke (66% vs 36%, respectively).^12,20–22^

Our findings of reduced social roles and participation after stroke are consistent with some,^23–25,27^ but not all previous studies.^28,29^ In agreement with previous studies,^23–25,27^ patients with previous stroke or TIA, and poor functional outcome at discharge, more often reported reduced social participation, but we additionally found associations with admission stroke severity and ICH which may be important to better target interventions, such as training carers, improved social support, dedicated rehabilitation, and treatment of depression.^30^

Our findings confirm that pain is commonly reported after stroke (overall prevalence 47.9%), consistent with previous estimates of between 11% and 55%.^26,31–33^ We found that pain is more common after ICH than ischemic stroke (60.3% vs 46%) consistent with a small cohort study which reported pain to be more frequent after thalamic ICH than ischemic stroke.^34^

We identified sleep disturbance in 40.9% of patients at 30-day follow-up, in agreement with previous studies reporting prevalence of 20–67% at 1 month to 5 years after stroke;^35–40^ black ethnic origin was associated with sleep disturbance, consistent with previous large cohort studies.^39,40^

We found that bladder dysfunction was more prevalent than bowel dysfunction and that bowel dysfunction was more prevalent after ICH than ischemic stroke, in line with a previous meta-analysis.^41^ We found a lower prevalence of bowel dysfunction than some previous reports, which could be attributed to not sub-classifying fecal incontinence and *s* constipation, underreporting, the screening tool we used (Barthel Index), or selection or ascertainment bias in other studies.^41–43^

Few previous studies have investigated patterns of co-occurrence and correlations between adverse non-motor outcomes after stroke. We found a high burden of multiple adverse non-motor outcomes, with about two-thirds of participants reporting two or more, and nearly on-half reporting three or more. We also found that all adverse non-motor outcomes were associated with the co-occurrence of one, two, or three others (i.e. none occurred in isolation), but that some co-occurred more often than expected by chance (anxiety and fatigue, social participation and bladder function, and bladder and bowel function).

Our study has strengths. We included consecutive patients with acute stroke from a defined ethnically diverse large and defined North London population, and systematically assessed adverse non-motor outcomes in multiple health domains. Stroke diagnosis was confirmed by brain imaging ensuring accurate diagnosis and classification. We used false discovery rate analysis to avoid false-positive findings and were able to assess other independent baseline predictors (including ICH) while adjusting for potential confounding factors.

We also acknowledge potential limitations including potential selection bias, though this was minimized by including sequential patients with a high rate of follow-up (84.1%). Compared to those included, there was a higher proportion of people of non-White ethnicity in those excluded, emphasizing the importance for future studies including all ethnic groups. We also acknowledge that for anxiety and depression PROMIS-29 measures symptom burden over the 7 days and does not provide a formal diagnosis of these conditions. Our data describe the burden on non-motor outcomes at 1 month, but more data are needed at longer-term follow-up. Although 47.8% of participants were discharged to an acute stroke unit (ASU), where they received rehabilitation input, including physiotherapy, speech and language, and occupational therapy, we do not have detailed data on the exact degree of rehabilitation input received for each participant. Furthermore, we did not have access to neuropsychological interventions provided after HASU discharge.

Our findings regarding the prevalence, patterns, and predictors of adverse patient-reported non-motor outcomes should help stroke services to plan pathways to first ascertain and then address these patient-reported adverse outcomes to improve post-stroke quality of life and provide patient-centered stroke care pathways. However, further long-term studies—including information on functional impact—are needed to fully establish the clinical relevance of these patient-reported non-motor outcomes.

## Supplemental Material

sj-docx-1-wso-10.1177_17474930231215660 – Supplemental material for Prevalence, patterns, and predictors of patient-reported non-motor outcomes at 30 days after acute stroke: Prospective observational hospital cohort study Supplemental material, sj-docx-1-wso-10.1177_17474930231215660 for Prevalence, patterns, and predictors of patient-reported non-motor outcomes at 30 days after acute stroke: Prospective observational hospital cohort study by Hatice Ozkan, Gareth Ambler, Gargi Banerjee, Simone Browning, Alex P Leff, Nick S Ward, Robert J Simister and David J Werring in International Journal of Stroke

## References

[1] GBD 2019 Stroke Collaborators. Global, regional, and national burden of stroke and its risk factors, 1990–2019: a systematic analysis for the global burden of disease study 2019. Lancet Neurol 2021; 20: 795–820. 34487721 10.1016/S1474-4422(21)00252-0 PMC8443449

[2] Braun RG Heitsch L Cole JW , et al. Domain-specific outcomes for stroke clinical trials: what the modified rankin isn’t ranking. Neurology 2021; 97: 367–377. 34172537 10.1212/WNL.0000000000012231 PMC8397584

[3] Douven E Köhler S Rodriguez MMF Staals J Verhey FRJ Aalten P . Imaging markers of post-stroke depression and apathy: a systematic review and meta-analysis. Neuropsychol Rev 2017; 27: 202–219. 28831649 10.1007/s11065-017-9356-2 PMC5613051

[4] Dennis M O'Rourke S Lewis S Sharpe M Warlow C . Emotional outcomes after stroke: factors associated with poor outcome. J Neurol Neurosurg Psychiatry 2000; 68: 47–52. 10601401 10.1136/jnnp.68.1.47 PMC1760616

[5] Maaijwee NA Arntz RM Rutten-Jacobs LC , et al. Post-stroke fatigue and its association with poor functional outcome after stroke in young adults. J Neurol Neurosurg Psychiatry 2015; 86: 1120–1126. 25362090 10.1136/jnnp-2014-308784

[6] Chen T Zhang B Deng Y , et al. Long-term unmet needs after stroke: systematic review of evidence from survey studies. BMJ Open 2019; 9: 28–137. 10.1136/bmjopen-2018-028137 PMC6530326 31110106

[7] van Mierlo ML van Heugten CM Post MW Hajós TR Kappelle LJ Visser-Meily JM . Quality of life during the first two years post stroke: the Restore4Stroke cohort study. Cerebrovasc Dis 2016; 41: 19–26. 26580841 10.1159/000441197

[8] Donnellan C Hickey A Hevey D O’Neill D . Effect of mood symptoms on recovery one year after stroke. Int J Geriatr Psychiatry 2010; 25: 1288–1295. 21086539 10.1002/gps.2482

[9] SNAPP UK . National clinical guideline for stroke: life after stroke, https://www.strokeguideline.org/contents/ (2023, accessed 13 July 2023).

[10] Olive-Gadea M Cano D Rodrigo-Gisbert M , et al. Redefining disability: patient-reported outcome measures after minor stroke and transient ischemic attack. Stroke 2023; 54: 144–150. 36300370 10.1161/STROKEAHA.122.040409

[11] Katzan IL Thompson N Schuster A , et al. Patient-reported outcomes predict future emergency department visits and hospital admissions in patients with stroke. JAHA 2021; 10: e018794. 10.1161/JAHA.120.018794 PMC8174209 33666094

[12] Hackett ML Köhler S O’Brien JT , et al. Neuropsychiatric outcomes of stroke. Lancet Neurology 2014; 13: 525–534. 10.1016/S1474-4422(14)70016-X 24685278

[13] De Wit L Theuns P Dejaeger E , et al. Long-term impact of stroke on patients’ health-related quality of life. Disabil Rehabil 2017; 39: 1435–1440. 27385479 10.1080/09638288.2016.1200676

[14] Zeng YY Cheng HR Cheng L , et al. Comparison of poststroke depression between acute ischemic and haemorrhagic stroke patients. Geriatr Psychiatry 2021; 36: 493–499. 10.1002/gps.5444 33108011

[15] Katzan IL Schuster A Newey C , et al. Patient-reported outcomes across cerebrovascular event types: more similar than different. Neurology 2018; 91: e2182–e2191. 10.1212/WNL.0000000000006626 30381370

[16] Thissen D Steinberg L Kuang D . Quick and easy implementation of the Benjamini-Hochberg procedure for controlling the false positive rate in multiple comparisons. JEBS 2002; 27: 77–83.

[17] Alghamdi I Ariti C Williams A , et al. Prevalence of fatigue after stroke: a systematic review and meta-analysis. ESJ 2021; 7: 23969873211047681. 10.1177/23969873211047681 PMC8948505 35342803

[18] Gallacher KI Jani BD Hanlon P , et al. Multimorbidity in stroke. Stroke 2019; 50: 1919–1926. 31233391 10.1161/STROKEAHA.118.020376

[19] McCaffrey N Kaambwa B Currow DC , et al. Health-related quality of life measured using the EQ-5D–5L: South Australian population norms. Health and quality of life outcomes. J Neurol 2016; 14: 1–2. 10.1186/s12955-016-0537-0 PMC5028927 27644755

[20] Cumming TB Packer M Kramer SF , et al. The prevalence of fatigue after stroke: a systematic review and meta-analysis. Int J Stroke 2016; 11: 968–977. 27703065 10.1177/1747493016669861

[21] Ayerbe L Ayis SA Crichton S Wolfe CD Rudd AG . Natural history, predictors, and associated outcomes of anxiety up to 10 years after stroke: the South London stroke register. Age Ageing 2014; 43: 542–547. 24375225 10.1093/ageing/aft208

[22] Paciaroni M Acciarresi M . Poststroke fatigue. Stroke 2019; 50: 1927–1933. 31195940 10.1161/STROKEAHA.119.023552

[23] Kossi O Nindorera F Adoukonou T Penta M Thonnard JL . Determinants of Social Participation at 1, 3, and 6 Months Poststroke in Benin. Arch Phys Med Rehabil 2019; 100: 2071–2078. 31029652 10.1016/j.apmr.2019.03.020

[24] Van Der Zee CH Visser-Meily JM Lindeman E , et al. Participation in the chronic phase of stroke. Top Stroke Rehabil 2013; 20: 52–61. 23340071 10.1310/tsr2001-52

[25] Blömer AM van Mierlo ML Visser-Meily JM van Heugten CM Post MW . Does the frequency of participation change after stroke and is this change associated with the subjective experience of participation. Arch Phys Med Rehabil 2015; 96: 456–463. 25264108 10.1016/j.apmr.2014.09.003

[26] O'Donnell MJ Diener HC Sacco RL , et al. Chronic pain syndromes after ischemic stroke: PRoFESS trial. Stroke 2013; 44: 1238–1243. 23559265 10.1161/STROKEAHA.111.671008

[27] Drummond A . Leisure activity after stroke. Int J Disabil Stud 1990; 12: 157–160. 10.3109/03790799009166608 2103567

[28] Vincent- Onabajo GO . Social participation after stroke: one-year follow-up of stroke survivors in Nigeria. Int Sch Res Notices 2013; 2013: 532518.

[29] Edwards DF Hahn M Baum C Dromerick AW . The impact of mild stroke on meaningful activity and life satisfaction. J Stroke Cerebrovasc Dis 2006; 15: 151–157. 17904068 10.1016/j.jstrokecerebrovasdis.2006.04.001

[30] Schnitzler A Jourdan C Josseran L Azouvi P Jacob L Genêt F . Participation in work and leisure activities after stroke: a national study. Ann Phys Rehabil Med 2019; 62: 351–355. 31096014 10.1016/j.rehab.2019.04.005

[31] Paolucci S Iosa M Toni D , et al. Prevalence and time course of post-stroke pain: a multicenter prospective hospital-based study. Pain Med 2016; 17: 924–930. 26814255 10.1093/pm/pnv019

[32] Klit H Finnerup NB Jensen TS . Central post-stroke pain: clinical characteristics, pathophysiology, and management. Lancet Neurol 2009; 8: 857–868. 19679277 10.1016/S1474-4422(09)70176-0

[33] Weimar C Kloke M Schlott M Katsarava Z Diener HC . Central poststroke pain in a consecutive cohort of stroke patients. Cerebrovasc Dis 2002; 14: 261–263. 12403962 10.1159/000065663

[34] Nagasaka K Takashima I Matsuda K , et al. Late-onset hypersensitivity after a lesion in the ventral posterolateral nucleus of the thalamus: a macaque model of central post-stroke pain. Sci Rep 2021; 7: 10316. 10.1038/s41598-017-10679-2 PMC5583363 28871156

[35] Khot SP Morgenstern LB . Sleep and stroke. Stroke 2019; 50: 1612–1617. 31043150 10.1161/STROKEAHA.118.023553 PMC6640639

[36] Cai H Wang XP Yang GY . Sleep disorders in stroke: an update on management. Aging Dis 2021; 12: 570–585. 33815883 10.14336/AD.2020.0707 PMC7990374

[37] Sterr A Kuhn M Nissen C , et al. Post-stroke insomnia in community-dwelling patients with chronic motor stroke: physiological evidence and implications for stroke care. Sci Rep 2018; 8: 8409. 29849087 10.1038/s41598-018-26630-y PMC5976765

[38] Glozier N Moullaali TJ Sivertsen B , et al. The course and impact of poststroke insomnia in stroke survivors aged 18 to 65 years: results from the psychosocial outcomes in stroke (POISE) study. Cerebrovasc Dis Extra 2017; 7: 9–20. 28161702 10.1159/000455751 PMC5346918

[39] Hasan F Gordon C Wu D , et al. Dynamic prevalence of sleep disorders following stroke or transient ischemic attack: systematic review and meta-analysis. Stroke 2021; 52: 655–663. 33406871 10.1161/STROKEAHA.120.029847

[40] Springer MV Lisabeth LD Gibbs R , et al. Racial and ethnic differences in sleep-disordered breathing and sleep duration among stroke patients. J Stroke Cerebrovasc Dis 2022; 31: 106822. 36244278 10.1016/j.jstrokecerebrovasdis.2022.106822 PMC9802657

[41] Li J Yuan M Liu Y Zhao Y Wang J Guo W . Incidence of constipation in stroke patients: a systematic review and meta-analysis. Medicine 2017; 96: e7225. 10.1097/MD.0000000000007225 PMC5484225 28640117

[42] Engler TM Dourado CC Amâncio TG Farage L de Mello PA Padula MP . Stroke: bowel dysfunction in patients admitted for rehabilitation. Open Nurs J 2014; 8: 43–47. 25419252 10.2174/1874434601408010043 PMC4238029

[43] Williams MP Srikanth V Bird M Thrift AG . Urinary symptoms and natural history of urinary continence after first-ever stroke—a longitudinal population-based study. Age Ageing 2012; 41: 371–376. 22321907 10.1093/ageing/afs009

