# Supplementary material for: Prevalence, patterns, and predictors of patient-reported non-motor outcomes at 30 days after acute stroke: Prospective observational hospital cohort study
Source: Int J Stroke. 2023 Dec 18;19(4):442–51. doi: 10.1177/17474930231215660 (PMC10964387; doi:10.1177/17474930231215660)
Supplement: sj-docx-1-wso-10.1177_17474930231215660 – Supplemental material for Prevalence, patterns, and predictors of patient-reported non-motor outcomes at 30 days after acute stroke: Prospective observational hospital cohort study [file sj-docx-1-wso-10.1177_17474930231215660.docx]

**Appendix File**

| Page 2 | Table 1. Non-motor outcome prevalence for ischaemic stroke *vs* Intracerebral haemorrhage (CH) |
| --- | --- |
| Page 3 | Table 2. Patient-reported outcome measures by stroke type |
| Page 4 | Table 3. Characteristics of patients lost to follow-up |
| Page 5 | Table 4. Characteristics of patients reporting multiple adverse non-motor outcomes |

**Table 1 – Non-motor outcome prevalence for ischaemic stroke vs ICH**

| **Non-Motor Domains** | **All Stroke**  **(N, %)** | **ischaemic stroke (N, %)** | **ICH**  **(N, %)** | **Difference**  **[95% Cl]** | **Uncorrected *P* Value** | ***P* Value after FDR** |
| --- | --- | --- | --- | --- | --- | --- |
| **PROMIS-29 Scale** | | | | | | |
| Anxiety^567^ | 323 (57.0%) | 271 (55.0%) | 52 (70.3%) | 15.3 [13.04 – 27.43] | **0.013** | **0.035** |
| Depression^566^ | 212 (37.5%) | 184 (37.3%) | 28 (38.7%) | 1.4 [0.21 – 1.71] | 0.865 | 0.865 |
| Fatigue^567^ | 298 (52.7%) | 250 (50.6%) | 48 (65.8%) | 15.2 [12.23 – 27.31] | **0.016** | **0.032** |
| Sleep Disturbance^565^ | 231 (40.9%) | 208 (42.2%) | 23 (31.9%) | 10.3 [7.02 – 19.32] | 0.099 | 0.132 |
| Social Roles and Activities^565^ | 278 (49.3%) | 232 (47.2%) | 46 (63.0%) | 15.9 [14.10 – 28.11] | **0.011** | **0.044** |
| Pain^564^ | 270 (47.9%) | 226 (46.0%) | 44 (60.3%) | 14.2 [11.22 – 26.53] | **0.023** | **0.037** |
| **Barthel Index Scale** | | | | | | |
| Bowel^605^ | 99 (16.4%) | 75 (14.5%) | 24 (27.3%) | 16.4 [10.41 – 21.16] | **0.003** | **0.024** |
| Bladder^605^ | 303 (50.2%) | 262 (50.9%) | 41 (46.1%) | 4.8 [0.6 – 6.22] | 0.402 | 0.459 |

N= Number; Cl= Confidence Interval; FDR= False Discovery Rate; ICH= Intracerebral haemorrhage

| **Outcome Variables** | **All Stroke**  **(N, %)** | **ischaemic stroke (N, %)** | **ICH**  **(N, %)** | **Difference**  **[95% Cl]** | ***P* Value** |
| --- | --- | --- | --- | --- | --- |
| **PROMIS-29 Scale** | | | | | |
| Physical Function^567^ | 219 (38.6%) | 178 (33.8%) | 41 (51.9%) | 18.1 [14.01 – 23.47] | **<0.001** |
| **Barthel Index Scale** | | | | | |
| Feeding^605^ | 177 (29.3%) | 155 (29.5%) | 22 (27.9%) | 1.6 [1.12 – 2.81] | 0.768 |
| Bathing^605^ | 284 (46.9%) | 243 (46.2%) | 41 (51.9%) | 5.7 [3.42 – 9.13] | 0.344 |
| Grooming^605^ | 136 (22.8%) | 112 (21.3%) | 24 (30.4%) | 9.1 [5.67 – 11.23] | 0.071 |
| Dressing^605^ | 207 (34.2%) | 171 (31.5%) | 36 (45.6%) | 14.1 [12.34 – 17.91] | **0.023** |
| Toilet Use^605^ | 267 (44.2%) | 224 (42.6%) | 63 (54.4%) | 11.8 [8.71 – 13.41] | **0.011** |
| Transfers (Bed to Chair)^605^ | 254 (41.9%) | 214 (40.7%) | 40 (50.6%) | 9.9 [7.17 – 14.21] | 0.095 |
| Mobility (On Level Surface)^605^ | 219 (41.7%) | 219 (41.7%) | 44 (55.7%) | 14.0 [9.61 – 18.29] | **0.019** |
| Stairs^605^ | 277 (45.8%) | 226 (43.4%) | 51 (60.7%) | 17.3 [14.81 – 19.53] | **0.041** |

**Table 2 – Patient-reported outcome measures by stroke type**

N= Number; Cl= Confidence Interval; ICH= Intracerebral haemorrhage

**Table 3 – Characteristics of patients lost to follow-up**

| **Characteristics** | **Patients excluded due to being lost to follow-up**  **N= 114** | **Patients included**  **N= 605** | ***P Value*** |
| --- | --- | --- | --- |
| Stroke Type (ischaemic stroke) | 107 (93.8%) | 521 (86.1%) | 0.087 |
| Age range (IQR) | 68.2 ± 15.1 | 72.0 ± 14.9 | 0.5035 |
| Female sex | 61 (53.5%) | 292 (48.3%) | 0.105 |
| **Ethnicity n (%)** | |  |  |
| White | 55 (48.2%) | 392 (66.4%) | 0.026 |
| Asian | 15 (13.6%) | 48 (8.1%) | … |
| Black | 13 (11.4%) | 28 (4.8%) | … |
| Other | 31 (27.2%) | 122 (20.7%) | … |
| **Medical history n (%)** | |  |  |
| Previous stroke/TIA | 34 (29.8%) | 208 (34.4%) | 0.161 |
| Hypertension | 77 (67.5%) | 407 (67.9%) | 0.784 |
| Congestive Heart Failure | 9 (7.8%) | 27 (4.5%) | 0.543 |
| Diabetes Miletus | 34 (29.8%) | 166 (27.7%) | 0.964 |
| AF | 23 (20.2%) | 132 (21.8%) | 0.302 |
| Smoking History | 41 (36.0%) | 208 (37.3%) | 0.847 |
| **Medication history n (%)** | |  |  |
| Thrombectomy | 4 (3.5%) | 32 (5.3%) | 0.737 |
| Thrombolysis | 28 (24.7%) | 124 (20.5%) | 0.628 |
| Antiplatelet | 61 (53.5%) | 340 (56.2%) | 0.354 |
| Anticoagulant | 28 (24.7%) | 142 (23.5%) | 0.728 |
| Antihypertensive | 83 (72.8) | 468 (77.7%) | 0.102 |
| Statin | 54 (47.4%) | 260 (42.9%) | 0.067 |
| Pre-Morbid mRS | 0 (0 – 1) | 0 (0 – 1) | 0.294 |
| Admission NIHSS | 6.3 (2 – 9) | 4 (2 – 8) | 0.116 |
| Discharge mRS | 3 (1 – 4) | 3 (1 – 4) | 0.182 |
| 30-day mRS | 2 (1 – 3) | 2 (1 – 3) | 0.754 |
| **Discharge Location n (%)** | |  |  |
| Home with ESD | 33 (28.9%) | 141 (24.4%) | 0.009 |
| ASU | 36 (33.6%) | 277 (47.8%) | … |
| Care Home | 2 (1.9%) | 5 (0.9%) | … |
| Home No ESD | 43 (40.2%) | 156 (26.9%) | … |

ICH= intracerebral haemorrhagic stroke; IQR= interquartile range; TIA= transient ischaemic attack; AF= arterial fibrillation; NIHSS= NIH stroke scale score; mRS= modified Rankin Scale; ESD= early supported discharge; ASU= acute stroke unit.

| **Characteristics** | **Patients with ≥2 adverse non-motor outcomes**  **(N= 364)** | **Patients with <2 adverse non-motor outcomes**  **(N= 241)** | ***P* Value** |
| --- | --- | --- | --- |
| Stroke Type (ischaemic stroke) | 309 (85.1%) | 201 (83.4%) | 0.130 |
| Age range (IQR) | 71.8 ± 15.5 | 70.6 ± 15.3 | 0.7182 |
| Female sex | 179 (49.3%) | 117 (48.6%) | 0.371 |
| **Ethnicity n (%) (n= 357 for multiple domains)** | |  |  |
| White | 225 (63.03%) | 154 (64.7%) | 0.307 |
| Asian | 29 (8.1%) | 14 (5.9%) | … |
| Black | 21 (5.9%) | 11 (4.6%) | … |
| Other | 82 (22.9%) | 59 (24.8%) | … |
| **Medical history n (%)** | |  |  |
| Previous stroke/TIA | 115 (31.7%) | 70 (29.1%) | 0.598 |
| Hypertension | 237 (65.8%) | 154 (64.7%) | 0.163 |
| Congestive Heart Failure | 18 (5.0%) | 15 (6.3%) | 0.291 |
| Diabetes Miletus | 100 (27.8%) | 64 (26.9%) | 0.767 |
| AF | 70 (19.3%) | 51 (21.2%) | 0.821 |
| Smoking History (n= 328 for multiple domains) | 109 (33.2%) | 71 (32.2%) | 0.882 |
| **Medication history n (%)** | |  |  |
| Thrombectomy | 13 (3.6%) | 11 (4.56%) | 0.345 |
| Thrombolysis | 73 (20.0%) | 48 (19.9%) | 0.561 |
| Antiplatelet | 201 (55.4%) | 138 (57.3%) | 0.124 |
| Anticoagulant | 78 (21.5%) | 53 (21.9%) | 0.145 |
| Antihypertensive | 276 (76.2%) | 180 (75.0%) | 0.424 |
| Statin | 162 (44.6%) | 119 (49.4%) | 0.261 |
| Pre-Morbid mRS | 0 (0 – 1) | 0 (0 – 1) | 0.6079 |
| Admission NIHSS | 7 (4 – 12) | 4 (3 – 6) | 0.0450 |
| Discharge mRS | 2 (1 – 4) | 1 (0 – 2) | 0.0230 |
| 30-day mRS | 1 (0 – 2) | 1 (0 – 2) | 0.3803 |
| **Discharge Location n (%)** | |  |  |
| Home with ESD | 80 (22.8%) | 61 (26.3%) | 0.072 |
| ASU | 173 (49.3%) | 104 (43.1% | … |
| Care Home | 0 | 5 (2.1%) | … |
| Home No ESD | 98 (27.9%) | 62 (26.7%) | … |

**Table 4 – Characteristics of patients reporting multiple adverse non-motor outcomes**

ICH= intracerebral haemorrhagic stroke; IQR= interquartile range; TIA= transient ischaemic attack; AF= arterial fibrillation; NIHSS= NIH stroke scale score; mRS= modified Rankin Scale; ESD= early supported discharge; ASU= acute stroke unit.
